# Virginia State Veterinary Medical Association

**Published:** 1897-02

**Authors:** Thomas M. Sweeny

**Affiliations:** Secretary


Secretary.

### VIRGINIA STATE VETERINARY MEDICAL ASSOCIATION.

THE semiannual meeting was called to order by the President, Dr. George C. Faville, in the Odd Fellow's Hall, Staunton, Va., on Tuesday, January 5, 1897, at 11.45 A.M. The roll-call showed a very good attendance. The minutes of the previous meeting were read and approved.

After the report of the various committees and Board of Censors had been received and acted upon Dr. Burkholder read a very able and interesting paper on "The Examination of Horses for Soundness,"[1] which was fully discussed by the author and Drs. Harbaugh, Niles, Roop, and Faville.

[1] Will appear in March number.

Drs. Niles and Faville reported several cases of "Aphthous Fever," which elicited a warm discussion.

Dr. Faville also reported two cases of "Tetanus," in which the antitoxin treatment was successful.

The President, Dr. Faville, addressed the Association, remarking in part that the standing of the profession is advancing and that the people appreciate veterinary medicine now more than they formerly did. The Association is in a flourishing condition and has accomplished more than any other similar body, although it is one of the youngest. The State Examining Board, office of State Veterinarian, the Act of Legislature relative to contagious diseases, are all the work of the Association. The President also remarked that the State Board of Health should have a veterinarian as an active member, as also should the local boards. Various cities have taken up the subject of tuberculosis, and will doubtless take action in the matter in the near future.

After a vote of thanks to Dr. Burkholder, the resident member, for courtesies shown, the Association adjourned to meet in Richmond on Wednesday, June 23, 1897. THOMAS M. SWEENY,
Secretary.

